# Dietary leucine supplementation minimises tumour-induced damage in placental tissues of pregnant, tumour-bearing rats

**DOI:** 10.1186/s12885-016-2103-x

**Published:** 2016-02-04

**Authors:** Bread Leandro Gomes Cruz, Priscila Cristina da Silva, Rebeka Tomasin, Andre Gustavo Oliveira, Lais Rosa Viana, Emilianne Miguel Salomao, Maria Cristina Cintra Gomes-Marcondes

**Affiliations:** Department of Structural and Functional Biology, Biology Institute, State University of Campinas, UNICAMP, CP 6109, Campinas, São Paulo 13083862 Brazil; Rua Monteiro Lobato, 255, Campinas, Zip code 13083862 Brazil

**Keywords:** Cancer, Nutritional supplementation, Leucine, Cell signalling, Protein synthesis, Protein degradation

## Abstract

**Background:**

The occurrence of cancer during pregnancy merges two complex, poorly understood metabolic and hormonal conditions. This association can exacerbate the conditions of both the mother and the foetus. The branched-chain amino acid leucine enhances cellular activity, particularly by increasing protein synthesis. This study aimed to analyse the modulatory effect of a leucine-rich diet on direct and indirect tumour-induced placental damage. This was accomplished by evaluating the expression of genes involved in protein synthesis and degradation and assessing anti-oxidant enzyme activity in placental tissues collected from pregnant, tumour-bearing rats.

**Results:**

Pregnant rats were either implanted with Walker 256 tumour cells or injected with ascitic fluid (to study the indirect effects of tumour growth) and then fed a leucine-rich diet. Animals in a control group underwent the same procedures but were fed a normal diet. On the 20^th^ day of pregnancy, tumour growth was observed. Dams fed a normoprotein diet showed the greatest tumour growth. Injection with ascitic fluid mimicked the effects of tumour growth. Decreased placental protein synthesis and increased protein degradation were observed in both the tumour-bearing and the ascitic fluid-injected groups that were fed a normoprotein diet. These effects resulted in low placental DNA and protein content and high lipid peroxidation (measured by malondialdehyde content). Decreased placental protein synthesis-related gene expression was observed in the tumour group concomitant with increased expression of genes encoding protein degradation-associated proteins and proteolytic subunits.

**Conclusions:**

Consumption of a leucine-rich diet counteracted the effects produced by tumour growth and injection with ascitic fluid. The diet enhanced cell signalling, ameliorated deficiencies in DNA and protein content, and balanced protein synthesis and degradation processes in the placenta. The improvements in cell signalling included changes in the mTOR/eIF pathway. In conclusion, consumption of a leucine-rich diet improved placental metabolism and cell signalling in tumour-bearing rats, and these changes reduced the deleterious effects caused by tumour growth.

## Background

Cancer is the second leading cause of disease-mediated death. The most common types of cancer in women are breast and cervical cancers [1, 2]. Neoplasias are difficult to control and treat because they are associated with several biochemical and metabolic changes. These changes increase overall metabolic consumption in the host, especially in the skeletal muscle, and decrease lean body mass. This culminates in reduced quality of life and decreased survival in affected patients.

Moreover, the prevalence of cancer during pregnancy is not rare, and the coexistence of these two complex metabolic and hormonal conditions can negatively impact the mother and foetus [3, 4]. To date, few studies have reported on the influence of pregnancy on cancer and vice versa [3, 4]. Recent publications have indicated that pregnancy offers a protective effect against cancer, but other reports suggest that pregnancy can accelerate the growth and development of neoplasias. Furthermore, there are difficulties associated with diagnosing early-stage tumours in pregnant women [5, 6]. Zemlickis and colleagues [7] found that patients diagnosed with breast cancer during pregnancy exhibited more advanced tumours and a higher incidence of metastasis compared to non-pregnant controls. King and colleagues [8] found a greater incidence of advanced stage tumours in patients who were pregnant. Pregnant patients with cancer can exhibit a more advanced evolution of the disease due to delays in diagnosis and because the hormonal and physiological changes that accompany pregnancy can result in a more aggressive disease response.

The endocrine changes associated with cancer evolution during pregnancy have not been well studied because of the many physiological adaptations that concomitantly occur in the maternal organism. These adaptations are primarily driven by the maternal/foetal unit known as the placenta [9, 10]. Thus, additional studies examining how neoplastic progression is affected by the complex alteration of hormonal homeostasis imposed by pregnancy are needed. The aim of the current study was to evaluate how tumour development affects cellular activity in the placenta and how these changes interfere with placental protein metabolism. Previous studies have also shown that humoural factors produced by tumours and/or host cells can indirectly interfere with placental and foetal integrity. The consumption of a leucine-rich diet can enhance cellular activity; as such, this study also aimed to analyse the modulatory effect of nutritional supplementation of leucine on tumour-induced placental damage. This was accomplished by evaluating the expression of genes involved in protein synthesis and degradation and by assessing anti-oxidant enzyme activity in placental tissues collected from pregnant tumour-bearing rats.

## Methods

### Animals

Sixty adult female Wistar rats were obtained from the Animal Care Center at the CEMIB (Multidisciplinary Center for Biological Research of UNICAMP) and housed in the experimental room located in the Laboratory of Nutrition and Cancer. The animals received food and water *ad libitum* and were maintained under controlled light and temperature conditions (12 h light-12 h darkness, 22 + 2 °C, and 60 % relative humidity). To obtain pregnant rats, inbred mating was performed using 4 females per 1 male. Mating was allowed to occur for 12 hours, and pregnancy was detected by assessing vaginal smears for the existence of sperm. The pregnant rats were subsequently distributed into 6 groups (minimum of 10 animals per group). Three groups were fed a control diet: a control group (C), Walker tumour-bearing group (W), and ascitic fluid-injection group (A). The remaining three groups were fed a leucine-rich diet: a leucine-fed control group (L), tumour-bearing group (LW), and ascitic fluid-inoculation group (LA). Each animal in the two tumour-bearing groups (W and LW) was subcutaneously injected with approximately 1 × 10^6^viable Walker 256 tumour cells on the second day of pregnancy. Each animal in the ascitic fluid injection groups (A and LA groups) was injected with ascitic fluid from the 9^th^ to the 20^th^ day of pregnancy [11]. All of the animals were weighed three times per week. At the end of the experiment (20^th^ day of pregnancy), the dams were sacrificed, and samples of placenta were collected, weighed and quickly frozen in liquid nitrogen. They were subsequently stored at −80 °C for later biochemical and molecular analysis. Fresh placental tissues were immediately used for protein and degradation assays. At the time of dam sacrifice, the foetuses were weighed and counted, and the number of foetal resorptions was recorded.

The experimental protocol used to conduct this research was approved by the Ethics Committee on Animal Experimentation of the Institute of Biology at the State University of Campinas (UNICAMP), protocol number 2071–1. Additionally, the care and handling of the tumour-bearing animals was in accordance with the rules of the United Kingdom Coordinating Committee on Cancer Research, an international organization [12].

### Diets

The semipurified diets used in this study were equivalent to diets produced by the American Institute of Nutrition (AIN-93G) [13] and contained the same amounts of protein, calories and lipids. The diets were normoproteic, isocaloric and normolipidic. The control diet (C) contained 18 % protein and was composed of 20 % casein (as a protein source); 39.7 % corn starch, 13.2 % dextrin, and 10 % sugar (as carbohydrate sources); 7 % soy oil (as a fat source); 5 % cellulose micro fibre (as a fibre source); 3.5 % salt mix; 1.0 % vitamin mix; 0.3 % cysteine and 0.25 % choline. The leucine-rich diet (L) also contained 18 % protein and was composed of the same amounts of casein, fat, fibre, salt, vitamin mix, cysteine and choline as the control diet, as well as 3 % leucine and 38.7 % corn starch, 12.2 % dextrin, and 9 % sugar (as carbohydrate sources). The control diet contained 1.6 % L-leucine. The leucine-rich diet contained 4.6 % L-leucine [1, 2].

### Biochemical and molecular analyses

#### Placental synthesis and protein degradation

Fresh placental tissues were removed and individually placed into tubes for perfusion with Krebs-Henseleit buffer (KHB) (110 mM NaCl, 25 mM NaHCO_3_, 3.4 mM KCl, 1 mM CaCl_2_, 1 mM KH_2_PO_4_, 1 mM MgSO_4_, 5.5 mM glucose, 0.01 % (w/v) bovine serum albumin, pH 7.4). The placentas were pre-incubated for 30 min at 37 °C with continuous shaking and gassing (95 % O_2_ + 5 % CO_2_) [14]. Following this, fresh KHB buffer solution supplemented with phenylalanine (75 mmol L-phenylalanine and 50 mCi L-[2,6-^3^H] phenylalanine/mL, Amersham) was added and incubated for an additional 2 h. Next, the placentas were removed from the medium, dried, weighed, homogenised in 30 % TCA (1:3 w/v), and centrifuged at 10,000 x g for 15 min at 4 °C. The pellet was washed twice with 10 % TCA to remove radioactivity from the supernatant and then suspended in 1 M NaOH and incubated at 40 °C for 30 min. Aliquots were analysed for protein content [15], and radioactivity was measured using a liquid scintillation β counter (Beckman LS 6000 TA, Fullerton, CA). The rate of placental protein synthesis was calculated as the amount of radioactive phenylalanine incorporated into the precipitated protein in 2 h and expressed as nanomoles of [^3^H]-phenylalanine per microgram of placental protein per hour [14].

Another sample of fresh placenta was used to analyse protein degradation, which was expressed as nmol of tyrosine released per microgram of protein per hour. The placenta sample was pre-incubated for 30 min at 37 °C with RPMI-1640 medium without phenol. Following this, the sample was incubated with KHB buffer supplemented with 130 μg/mL cycloheximide (a protein synthesis inhibitor) for 2 h at 37 °C and then gassed with 5 % CO2 + 95 % O2. Next, an aliquot of the incubation medium was assessed for tyrosine content, which was measured fluorometrically using 1-nitroso-2-naphthol and nitric acid (20 %) according to a method published by Waalkes & Udenfriend [16].

#### Analysis of placental DNA content and enzyme activity

Placental tissue was homogenised in homogenisation buffer (0.1 M NaCl; 10 mM EDTA; 0.03 M Tris–HCl, pH 8.0; 0.2 M sucrose and 0.01 % SDS) and incubated for 1 h at 65 °C. Subsequently, 8 M potassium acetate was added, followed by incubation and centrifugation under refrigeration. DNA was extracted from the upper phase using phenol:chloroform:isoamyl alcohol (25:24:1) and then precipitated with 100 % ethanol overnight at −80 °C. DNA content was quantified by measuring the 260/280 nm absorbance ratio using a spectrophotometer. The coefficient of variation (CV) ranged from 6.5 % to for the control, tumour and ascitic fluid samples.

Aliquots of placental homogenate supernatant were analysed for alkaline phosphatase (AP) activity using 37 mM 4-nitrophenyl disodium phosphate (p-NPP; Sigma). AP activity was expressed in nmol/min/mg protein [17]. For this assay, the CV ranged from 10.3 % to 20.8 % among all experimental groups. Glutathione S-transferase (GST) activity was assessed in the placental samples based on the conjugation of 1-chloro-2,4-dinitrobenzene (CDNB; Sigma) with glutathione. The activity was expressed as nmol/μg protein/min and calculated using an extinction coefficient of 9.6 as previously described [17] (the CV was ≤24.7 %). Placental glutathione reductase was measured following the method described in our previous work [17] (the CV was ≤14.1 % among the groups). The lipid peroxidation product malondialdehyde (MDA) was quantified by incubating the samples with n-methyl-2-phenylindole (MPO; Sigma) and then reading the absorbance at 590 nm. The results are expressed as nmol/μg protein [18], and the CV ranged up to 18.1 % for all samples.

#### Measuring the expression of placental protein synthesis-associated and protein degradation-associated genes by quantitative RT-PCR

Samples of placental tissue (10 mg) were lysed in 1 mL TRI reagent solution (Sigma Aldrich, Co, Ltd.; Dorset, UK), and total RNA was extracted as described by the manufacturer. The RNA concentration was determined based on the absorbance at 260 nm. cDNA was synthesised from 3 μg of placental RNA in a volume of 20 μL using MultiScribe™ Reverse Transcriptase and random hexamer primers (High Capacity cDNA Reverse Transcription Kits; Applied Biosystems, Foster City, CA, USA). To amplify cDNA using real-time PCR, reactions were performed in a total volume of 20 μL containing 5 mM each primer, 4 μL cDNA (20x diluted), 4 μL milli-Q H2O and 10 μL Fast SYBR Green 2X Master Mix (Applied Biosystems, Foster City, CA). The placenta samples were denatured at 95 °C for 10 min, followed by 40 PCR cycles at 95 °C/60 °C. PCR amplification was performed in triplicate. Quantitative PCR assays were performed using a StepOne Real Time System (Applied Biosystems, Foster City, CA). The purity of the amplified PCR products was verified based on melting curves. Target gene expression was analysed using 7500 Fast Real-Time PCR software, version 2.0.5 (Applied Biosystems, Foster City, CA), and the values are expressed relative to control levels (2^-ΔΔCt^). We analysed the expression levels of the protein degradation-associated and protein synthesis-associated genes as described in Table 1. To confirm the integrity of the extracted RNA, transcription of the housekeeping gene glyceraldehyde-3-phosphate dehydrogenase (GAPDH) (NM_017008.4) was used as an internal control.

**Table 1:** Detailed primers used to access the protein degradation and synthesis processes in placenta tissue

Protein degradation -associated			
Gene		Forward	Reverse
PC5	NM_053590.1	GGACTTGATGAAGAAGGAAAGG	GATGCCCTCTTTGGTCACTATG
PC2	NM_017278.1	CAGGAGTGTTTGGATTCCAG	GTTAGCAGATGGACAGGTTTGG
Murf-1	NM_080903	TGAAGTGATCATGGACCGGC	AATGCTCTTGATGAGCGGCTT
Atrogin	NM_133521	ATGCCGTTCCTTGGTCAGG	ATGGCGCTCCTTAGTACTCCC
Ubiquitin	NM_031138.2	GAGGCTCATGCGGGATTTCAAG	TCCTGATAAAGCTGTGCTGC
Calpain	AB384822.1	GAACCGCATCCGGAATTACCTG	GGTTGCAGCTGACCATGTTTGCA
Protein synthesis-associated genes			
mTOR	NM_001134499.2	CTCGTGAAGGACAACGGTCA	TAGAGTTTTTCGTGGGCGCT
p70S6K1	NM_031985	GGCCCTGGGGATGCTGGAGA	GGCTGACAGGCGTTCGTGGG
eIF4E	NM_053974	ACCCCTACCACTAATCCCCC	GCTTCAGTGCAGTCCACTCT
eIF4G	NM_013507.3	ACCCACAATGGGACGTCATC	CCGAGGTGGCATATCCTTCG
4EBP1	NM_053857.2	CGGCACGCTCTTCAGCACCA	ACACCCAGTGTCTGCCGGGT
eIF5	XM_001479599.1	TGTCTGTCAACGTCAACCGCAG	GAGGCATGCTTGACACACATC
eIF2α	AK312442.1	GATCCATTGCTGAAATGGGG	GATGACAAGTACAAGAGACCTGG

#### Western blotting to determine placental protein expression

Placenta samples were homogenised in protein extraction buffer (100 mM Tris Base, 10 mM Na_4_P_2_O_7_, 100 mM FNa, 1 mM Na_3_ VO_4_, 10 mM EDTA, 2 mM PMSF, 0.1 mg/mL aprotinin, 1 % Triton X-100, pH 7.4) followed by centrifugation at 10,000 × g for 15 min at 4 °C. Placental proteins (40 μg) were resolved by 12 % SDS-PAGE at 90 V for 1 h, followed by transfer onto 0.45-μm nitrocellulose membranes at 300 mA for 2 h. The membranes were blocked with 5 % non-fat dry milk in Tris-buffered saline (pH 7.5) for 1 h at room temperature. The membranes were incubated overnight at 4 °C with primary antibodies against the 20S, 19S and 11S proteasome subunits (#PW8165, #PW8195, Affinity, USA; 1:1500 dilution) as well as MuRF-1 (#SC-32920) and MAFbx (#SC-33782; both from Santa Cruz Biotechnology, Heidelberg, Germany; 1:200 dilution). Immunoreactivity was detected by sequentially incubating the membranes with specific secondary antibodies (1:10,000 dilution, Cell Signaling Technology) for 1 h at room temperature and visualised using a chemiluminescence detection system. The blots were scanned using a gel image capture system to quantify differences via densitometry (Alliance 2.7 system, Alliance 1D capture software, and UVIBand 12.14 analysis software; UVITEC, Cambridge, UK). The levels of all detected proteins were reported relative to the level of 34-kDa GAPDH.

### Statistical analysis

The results are expressed as the mean ± S.E.M. All data were assessed using the Kolmogorov-Smirnov normality test, and the effects of diet, tumour presence, and ascitic fluid on placental and foetal parameters were statistically analysed by two-way ANOVA (Graph Pad Prism software, version 5.0, San Diego, CA). Inter-group comparisons were made using the post hoc Bonferroni multiple-comparison test. The results were considered significant when *P* < 0.05 [19].

## Results

### Tumour growth deleteriously affects dam body weight and foetal parameters

**Fig. 1:**
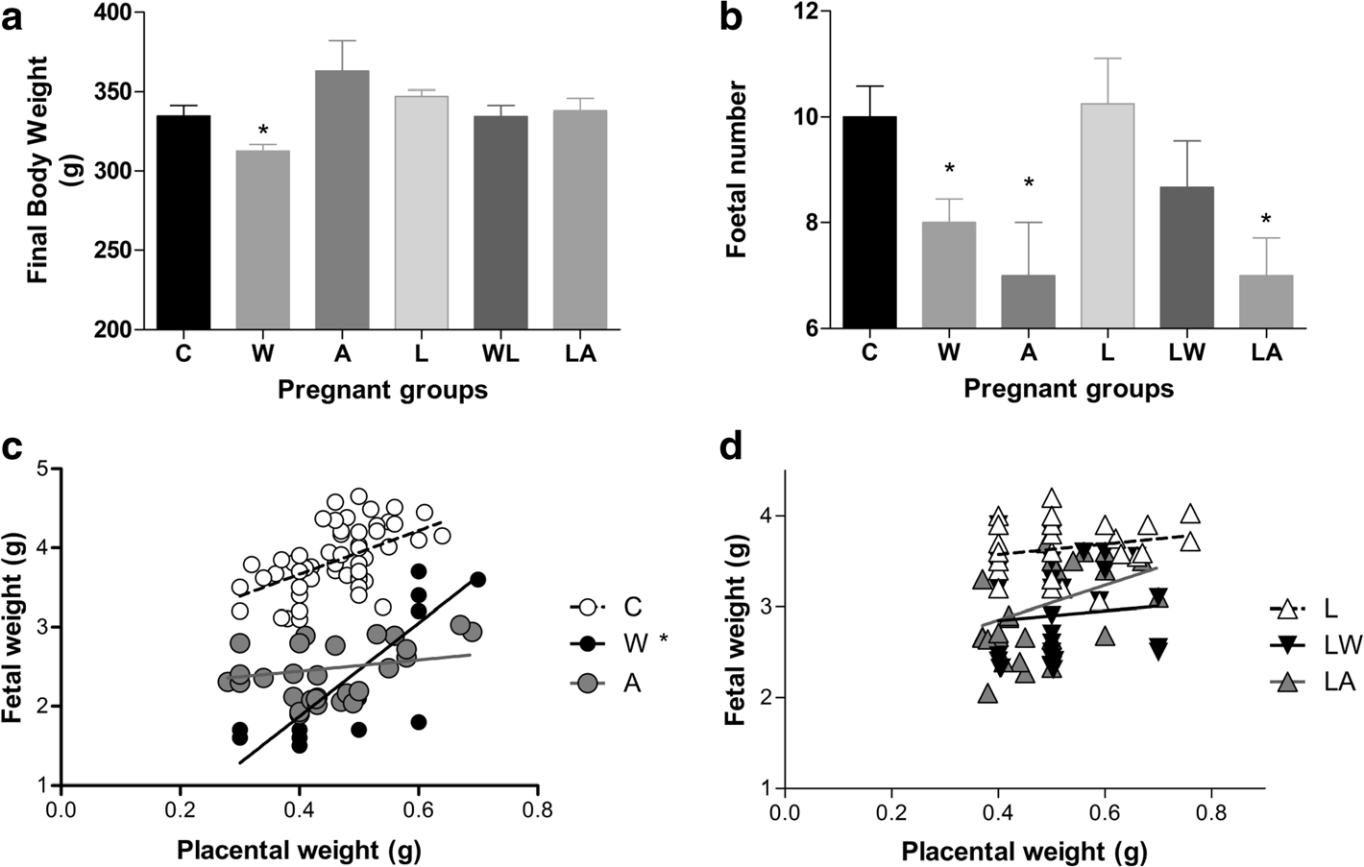
Body weight (**a**), foetal number (**b**), and foetal/placental weight ratio (**c**, eutrophic groups; **d**, groups fed a leucine-rich diet) in pregnant rats of different experimental groups. Legend: Control (C), Leucine (L), Walker tumour-bearing rats (W), tumour-bearing rats fed a leucine-rich diet (LW), ascitic fluid-injected rats (A) and ascitic fluid-injected rats fed a leucine-rich diet (LA). The results are expressed as the mean and standard error. * *P* < 0.05 accounted by tumour effect, two-way ANOVA with Bonferroni testing, *n* = 10. Graphic C shows individual data corresponding to foetal and placenta tissues obtained from each rat in each group; the C group curve has a slope of 2.61 slope for 74 analysed points. The W group curve has a slope of 16.7 for 32 analysed points and is significant (*P* < 0.05) based on two-way ANOVA with Bonferroni testing (*n* = 10). The A group curve has a slope of 0.92 for 38 analysed points. By linear regression analysis, it showed significant difference between slopes, *F* = 39.7, *P* < 0.0001. Graphic D shows individual data corresponding to foetal and placenta tissues obtained from each rat in each group; the L group curve has a slope of 0.70 for 87 analysed points. The LW group curve has a slope of 1.55 for 33 analysed points. The LA group curve has a slope of 2.07 for 36 analysed points. Linear regression analysis showed no difference among slopes (*F* = 0.19, *P* = 0.819)

Tumour growth produced harmful effects on body weight evolution in the W group: a significant variation of 31 % was observed (*P* < 0.0045). However, injections with ascitic fluid had no effect on body weight. Additionally, regardless of tumour presence, the leucine-treated group (WL) exhibited better body weight recovery compared to the C and L groups (Fig. 1a). In this case, the interaction between tumour, ascitic fluid and diet accounted for 20 % of the total variance and was highly significant (*P* < 0.0234). As in our previous experiments, a significant tumour effect leading to a reduction in the number of foetuses was observed (Fig. 1b, *P* = 0.0043). Additionally, tumour-bearing, pregnant animals exhibited increased foetal resorption and reduced foetal weight (Table 2; 38 % of total variance; *P* < 0.0047). Similarly, injections with ascitic fluid resulted in decreased foetal number and weight and increased foetal resorption (Fig. 1b and Table 2; the ascitic effect accounted for 36 % of the total variance, *P* < 0.0001). Tumour-bearing rats fed a leucine-rich diet (WL) exhibited a non-significantly decreased number of foetuses and increased foetus resorption relative to the control group (C); however, average foetal weight was significantly reduced compared to the C and L groups. Consumption of a leucine-rich diet minimised the effects caused by ascitic fluid injection: foetal resorption rate were maintained. The interaction between ascitic fluid and diet accounted for 20 % of the variation, which was significant (*P* < 0.0321); however, the diet could not correct for low foetal weight. The relationship between foetal weight and placental weight is indicative of the efficiency of placental transport (Fig. 1c and d). Foetal weight and placental weight were positively correlated in all groups except the tumour-bearing group (W). This correlation indicates that foetal and placental weights were not altered in parallel. As shown in the graph in Fig. 1c, variations in placenta weight resulted in low foetal weight; the linear regression analysis showed the difference between slopes was extremely significant (*F* = 39.7, *P* < 0.0001). In contrast, in the LW group, there was a positive correlation between placental and foetal weight. This relationship indicated that placental weight influenced foetal weight in this group (Fig. 1d; identical slopes, *F* = 0.19, *P* = 0.819).

**Table 2:** Foetal and placental parameters in Walker tumour-bearing rats and ascitic fluid-injected rats fed either a control or leucine-rich diet

	C	W	A	L	LW	LA	Interaction effect^b^	Tumour effect^b^	Diet effect^b^
Foetal resorption per dam	0_._900_±_0_._314	3_._300±0_._538*	2_._500±0_._562*	0_._889±0_._309	1_._600±0_._499	1_._700±0_._473	*P* = 0.0321	*P* = 0.0043	n.s.
Foetal weight (g)	3.92 ± 0.07	2.73 ± 0.20*	2.61 ± 0.05*	3.65 ± 0.06	2.85 ± 0.08*	2.96 ± 0.09*	n.s.	*P* < 0.0001	n.s.
Placenta weight (g)	0.48 ± 0.01	0.53 ± 0.02	0.42 ± 0.02	0.52 ± 0.03	0.52 ± 0.01	0.50 ± 0.02	n.s.	n.s.	n.s.
Placental protein content (μg/mg)	7.38 ± 0.48	3.81 ± 0.29*	4.70 ± 0.34*	7.14 ± 0.28	6.87 ± 0.56	6.37 ± 0.67	*P* = 0.0043	*P* = 0.0002	n.s.
Placental DNA (μg/mg)	0.46 ± 0.02	0.38 ± 0.02*	0.38 ± 0.02*	0.46 ± 0.03	0.49 ± 0.02	0.43 ± 0.04	n.s.	*P* = 0.0052	n.s.
Placental protein/DNA ratio^a^	16.8 ± 1.2	11.5 ± 0.9*	14.2 ± 1.5	17.3 ± 1.1	15.4 ± 1.5	17.3 ± 2.5	*P* = 0.0212	*P* = 0.0311	n.s.

Caption: Control (C), Leucine (L), Walker tumour-bearing rats (W), tumour-bearing rats subjected to a leucine-rich diet (LW), rats inoculated with ascitic fluid (A), and rats inoculated with ascitic fluid and subjected to a leucine-rich (LA). The results are expressed as the mean and standard deviation. *P < 0.05 compared with the control group. ^a^Placental protein/DNA ratio was used as an index of cell size. ^b^ Significant difference accounted by tumour, diet or interaction effect, analysed by two-way ANOVA with Bonferroni testing, n.s. = non-significant

### Placental tissue damage caused by tumour growth

**Fig. 2:**
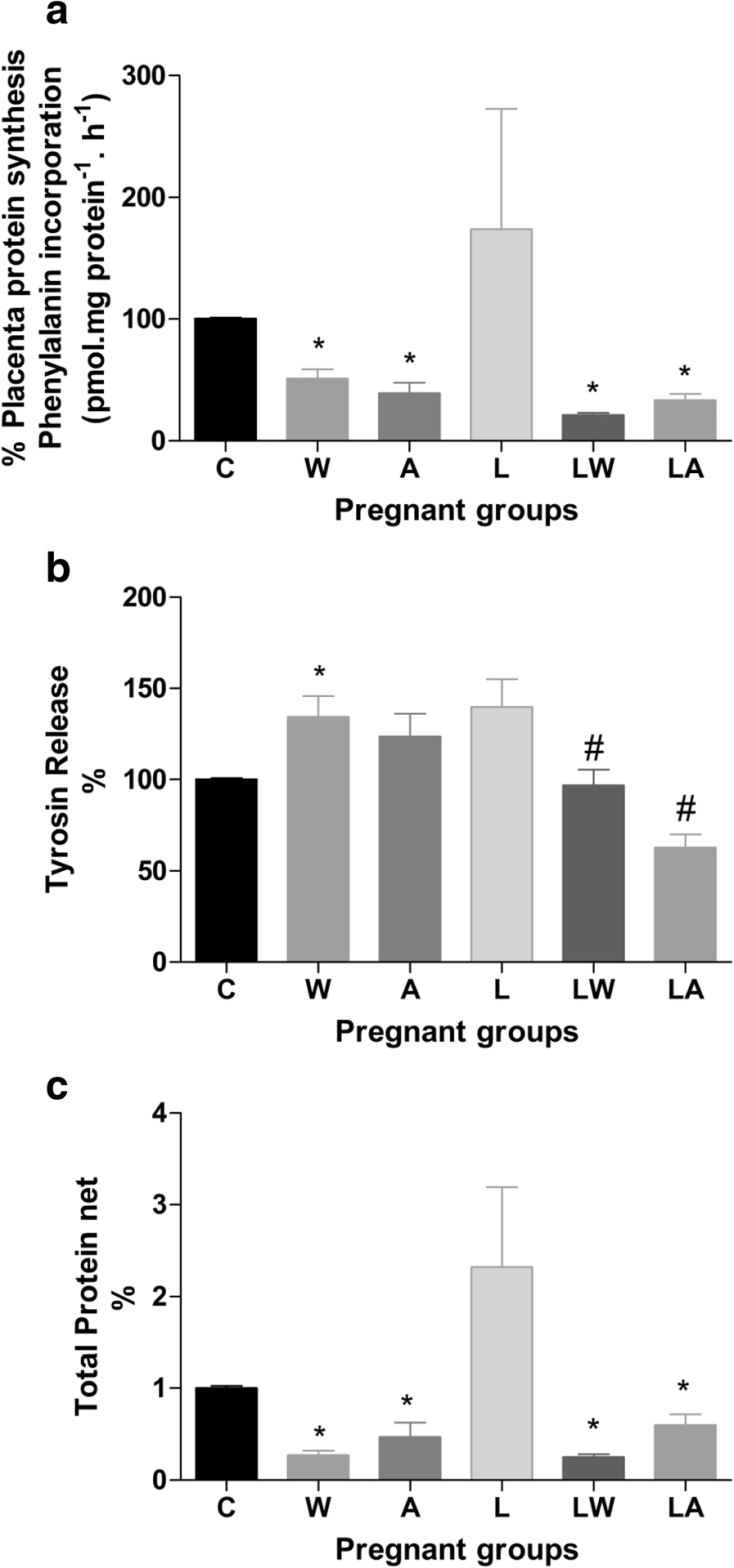
Placental protein synthesis (**a**), protein degradation (**b**) and protein balance (**c**) in pregnant rats from different experimental groups. Legend: Control (C), Leucine (L), Walker tumour-bearing rats (W), tumour-bearing rats fed a leucine-rich diet (WL), ascitic fluid-injected rats (A) and ascitic fluid-injected rats fed a leucine-rich diet (LA). The results are expressed as the mean and standard error. For further details, see the *Materials and Methods* section. * *P* < 0.05 accounted by tumour effect, two-way ANOVA with Bonferroni testing, *n* = 10. # *P* < 0.05 accounted by interaction between tumour/ascitic with diet effect, two-way ANOVA with Bonferroni testing, *n* = 10

Placental weights were similar among the groups. However, in the tumour-bearing and ascitic fluid-injected groups, placental protein content, DNA content, and protein/DNA ratio, which represent cell number, all decreased (the effects were significant, *P* < 0.005; Table 2). The same trends were not found in the leucine-rich diet groups; the interaction effect was very significant, but the diet had no effect overall (Table 2, *P* < 0.005). In addition to the above tumour effects, placental protein synthesis decreased in both the tumour-bearing (W) and ascitic fluid-injected groups (A) (Fig. 2a). The tumour and ascitic effects accounted for 15.0 % of the total variance and were significant (*P* = 0.0128). Consumption of a leucine-rich diet significantly increased placental phenylalanine incorporation in the L group (*P* = 0.0452) but either had no effect or reduced phenylalanine incorporation in the LW and LA groups (Fig. 2a). The tumour-bearing group (W) exhibited increased protein degradation; the tumour effect was significant at *P* = 0.0378 (Fig. 2b). Consumption of a leucine-rich diet increased placental tyrosine release in the L group. In the LW group, placental tyrosine release was reduced compared with that in the W group. The LA group exhibited a significant decrease, as the interaction effect between tumour/ascitic fluid and diet was very significant (*P* = 0.0003, Fig. 2b). Total protein levels in the placenta were very significantly affected by the presence of tumours/injections with ascitic fluid (15 % of total variance, *P* = 0.0018). This result indicates reduced balance of total protein net in the tumour-bearing and ascitic fluid-injected groups (Fig. 2c).

**Fig. 3:**
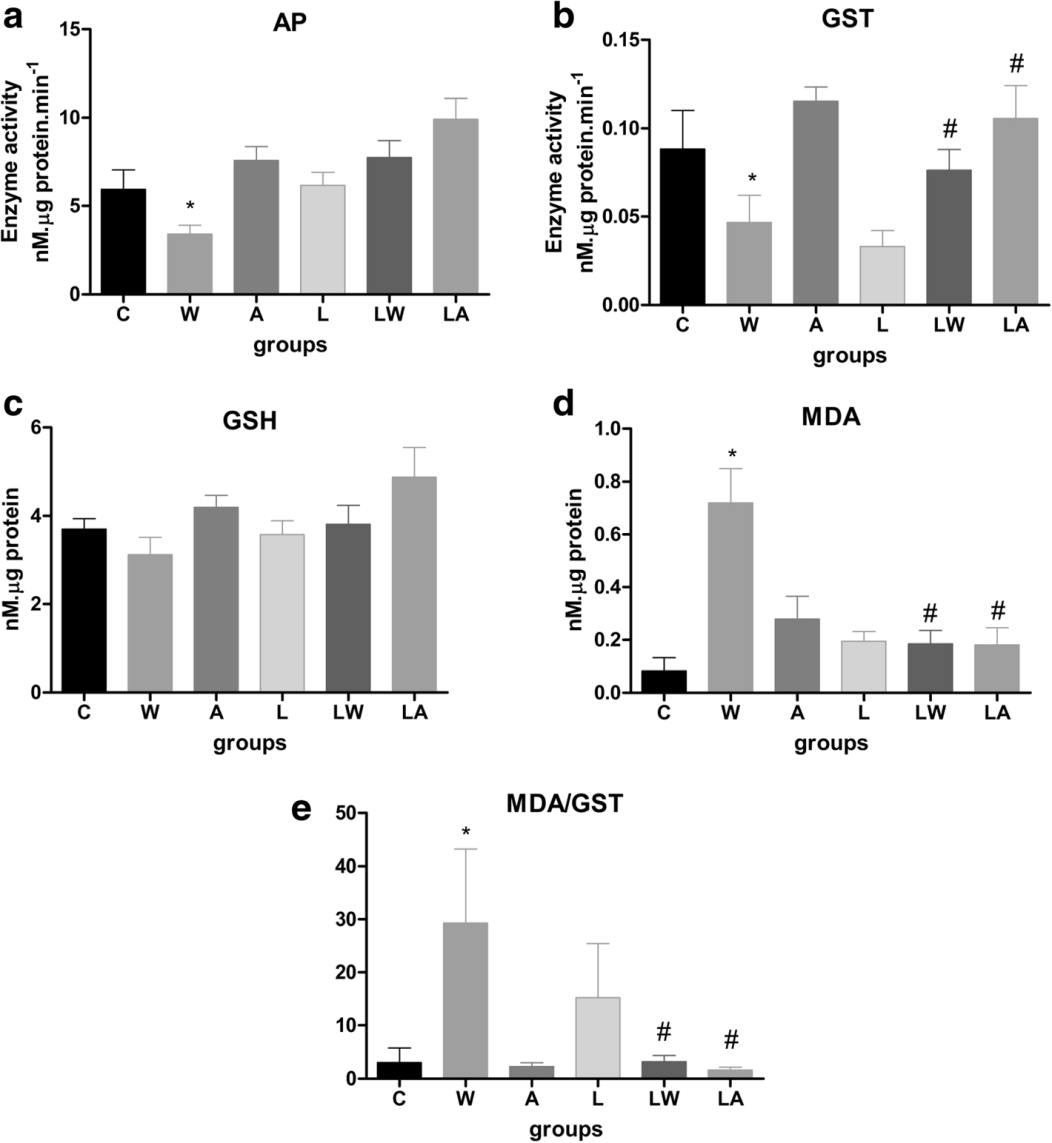
Activities of alkaline phosphatase (**a**), glutathione S-transferase (**b**), and reduced glutathione (**c**) in the placenta. Placental MDA content (**d**) and MDA/GST ratios (**e**) in pregnant rats from different experimental groups. Legend: Control (C), Leucine (L), Walker tumour-bearing rats (W), tumour-bearing rats fed a leucine-rich diet (WL), ascitic fluid-injected rats (A) and ascitic fluid-injected rats fed a leucine-rich diet (LA). The results are expressed as the mean and standard error. * *P* < 0.05 accounted by tumour effect, two-way ANOVA with Bonferroni testing, *n* = 10. # *P* < 0.05 accounted by interaction between tumour/ascitic with diet effect, two-way ANOVA with Bonferroni testing, *n* = 10

Overall cellular activity can be correlated with AP activity [17, 20]. Tumour growth decreased placental enzyme activity only in the W group (Fig. 3a, *P* = 0.003). Placental glutathione-S-transferase (GST) activity was only reduced in W group tumour-bearing dams (accounting for 28 % of total variance, *P* = 0.0019). Consumption of a leucine-rich diet significantly increased placental GST activity in the LW and LA groups versus the L group (Fig. 3b; interaction effect accounted for 16 % of variation, *P* = 0.019). The reduced glutathione levels were constant among the groups (Fig. 3c). Oxidative stress causes tissue damage, including lipid peroxidation and MDA release [18]. Oxidative damage has been associated with tumour growth. Placental tissue from the W group exhibited increased MDA content. In this case, the tumour effect accounted for 27 % of total variance and was extremely significant (Fig. 3d, *P* = 0.0005). Additionally, the interaction between tumour/ascitic fluid and diet produced an extremely significant effect (Fig. 3d, *P* = 0.0004). The MDA/GST ratio is an indicator of damage induced by poor cellular responses to external effects. The W group exhibited a significantly higher MDA/GST ratio relative to the other groups; the tumour effects in this group accounted for the significant difference (*P* = 0.0065). Such differences were not observed in the leucine-treated tumour-bearing or ascitic fluid-injected groups, in this case the interaction between tumour/ascitic fluid and diet had a significant effect (Fig. 3e, *P* = 0.0368).

**Fig. 4:**
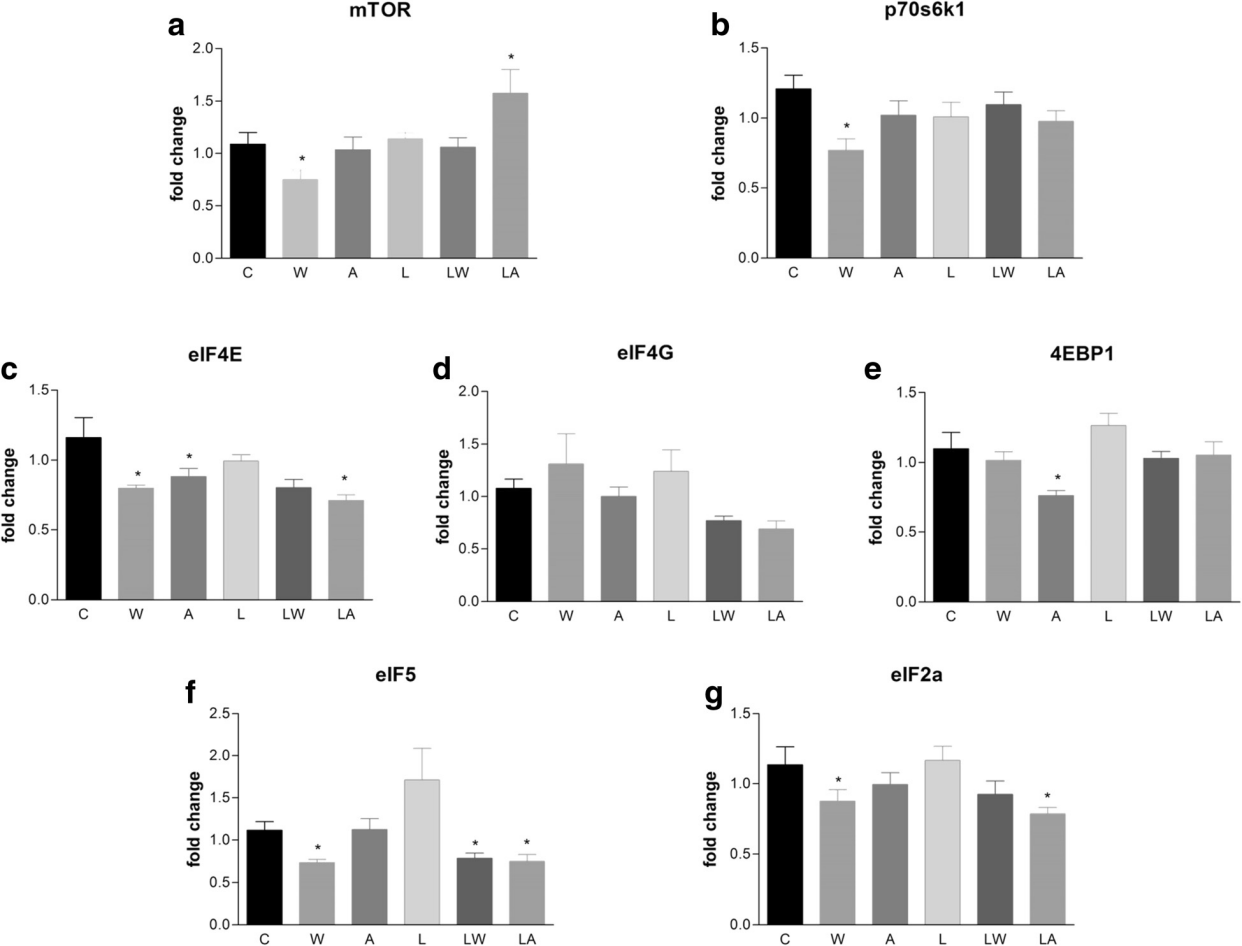
Protein-synthesis gene expression in placental tissues of pregnant, Walker tumour-bearing rats and ascitic fluid-injected rats fed either a control or leucine-rich diet. Mammalian target of rapamycin (mTOR) (**a**), p70-S6 kinase (p70S6K1) (**b**), eukaryotic initiation factors eIF4E (**c**) and eIF4G (**d**), eukaryotic translation initiation factor 4 binding protein 1 (4EBP1) (**e**), eIF5 (**f**) and eIF2a (**g**) transcript levels in placental tissue quantified by real-time PCR. Gene expression corrected by GAPDH as internal control. For additional details, see the *Materials and Methods* section. The results are expressed as the mean and standard error. Legend: Control (C), Leucine (L), Walker tumour-bearing rats (W), tumour-bearing rats fed a leucine-rich diet (WL), ascitic fluid-injected rats (A) and ascitic fluid-injected rats fed a leucine-rich diet (LA). * *P* < 0.05 accounted by tumour effect, two-way ANOVA with Bonferroni testing, *n* = 10

Tumour growth was associated with decreases in mTOR and p70S6K1 gene expression, as well as reduced expression of eIF4E, eIF5 and eIF2a, three factors involved in protein synthesis (Fig. 4a, b, c, f and g; the tumour effect was highly significant at *P* < 0.05). Ascitic fluid injection (A group) mimicked the indirect effects of tumour growth, including reductions in the expression of eIF4E and 4EBP1 (Fig. 4c and e; the ascitic fluid accounted for significant effects at *P* = 0.0504 and *P* = 0.0144, respectively). The leucine-supplemented groups (LW and LA) exhibited similar profiles that accounted for tumour/ascitic effects (10 % of the total variance; *P* = 0.0144) (Fig. 4c, d, f and g).

**Fig. 5:**
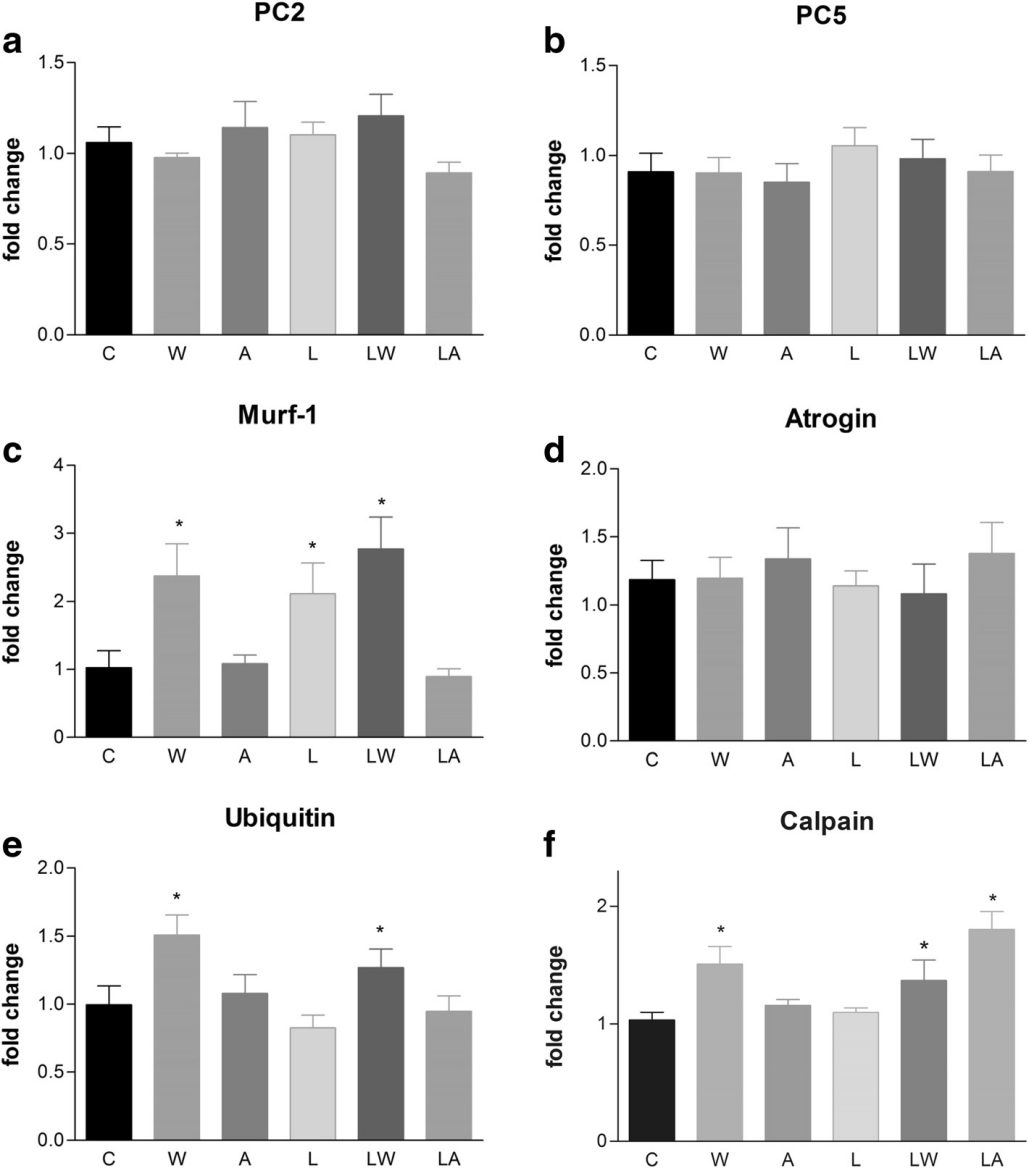
Protein-degradation gene expression in placental tissues of pregnant, Walker tumour-bearing rats and ascitic fluid-injected rats fed either a control or leucine-rich diet. Ubiquitin-proteasome pathway members, including the 20S subunit, represented by the PC2 (**a**) and PC5 (**b**) genes; muscle-ring finger protein 1 (MuRF-1) (**c**); ubiquitin ligase atrogin-1/MAFbx (**d**); ubiquitin (**e**); and a member of the calcium-dependent proteolytic pathway, calpain (**f**), were quantified in placental tissue using real-time PCR. Gene expression corrected by GAPDH as internal control. For additional details, see the *Materials and Methods* section. The results are expressed as the mean and standard error. Legend: Control (C), Leucine (L), Walker tumour-bearing rats (W), tumour-bearing rats fed a leucine-rich diet (WL), ascitic fluid-injected rats (A) and ascitic fluid-injected rats fed a leucine-rich diet (LA). * *P* < 0.05 accounted by tumour effect, two-way ANOVA with Bonferroni testing, *n* = 10

**Fig. 6:**
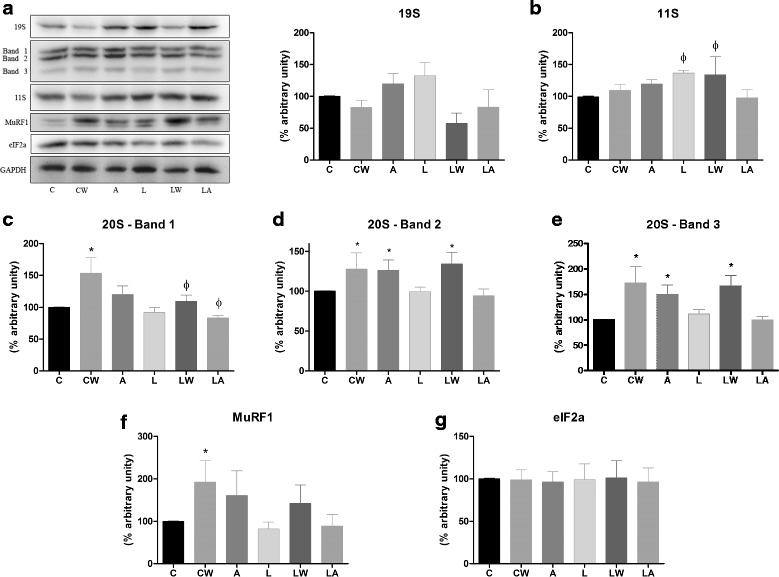
Key proteins involved in proteolysis in the placental tissues of pregnant, Walker tumour-bearing rats and ascitic fluid-injected rats fed either a control or leucine-rich diet. Expression levels of ubiquitin-proteasome pathway members, including the 19S (**a**), 20S (**b**, **c**, and **d**) and 11S subunits; (**e**), muscle-ring finger protein 1 (MuRF-1) (**f**); and the eukaryotic initiation factor eIF2a (**g**) in placenta tissue was quantified by western blotting. For additional details, see the *Materials and Methods* section. The results are expressed as the mean and standard error. The images represent one of 6 animals per group. Legend: Control (C), Leucine (L), Walker tumour-bearing rats (W), tumour-bearing rats fed a leucine-rich diet (WL), ascitic fluid-injected rats (A) and ascitic fluid-injected rats fed a leucine-rich diet (LA). * *P* < 0.05 accounted by tumour effect, two-way ANOVA with Bonferroni testing, *n* = 10. ϕ *P* < 0.05 accounted by diet effect, two-way ANOVA with Bonferroni testing, *n* = 10

Protein degradation also increases during tumour growth. Several key proteins were highly expressed in the placental tissues of the animals in the W group. These included MuRF-1, ubiquitin, ubiquitin-proteasome pathway enzymes, and calpain, which is related to the calcium-dependent proteolysis pathway. Tumour growth was responsible for highly significant effects (Fig. 5c, e and f; *P* = 0.027, *P* = 0.0147 and *P* = 0.0216). Similarly to the tumour/ascitic effect, we also observed increased expression of the MuRF-1 and ubiquitin genes in the LW group (Fig. 5c and e) and increased calpain gene expression increased in the LA group. Expression of the 20S and MuRF-1 proteins also increased, especially in the tumour-bearing groups (W and LW groups; Fig. 6b, c, d and f; tumour growth accounted for a significant effect at *P* = 0.0266). We also observed increased 11S protein expression in the L and LW groups, which were fed a leucine-rich diet; the effect of the diet was significant (Fig. 6e; *P* = 0.0509). No differences were observed among the groups with respect to 19S and eIF2a (Fig. 6a and g).

## Discussion

Tumour development, especially development of Walker 256 tumours, which serve as an experimental model of cachexia [21, 22], promotes the breakdown of structural tissue, including skeletal muscle mass [23], and increases the production of protein waste in the host. Tumour growth also affects other tissues and metabolic processes [24, 25]. In our previous experimental studies [20, 26–28] and in the present study, we found that tumour growth causes severe damage during the course of pregnancy. This damage is particularly harmful to placental tissue and results in significant foetal weight loss and increased foetal resorption [17, 20, 28]. Our previous results indicated that supplementation with a leucine-rich diet could prevent some of the effects induced by tumour evolution. Consumption of a leucine-rich diet also minimised the indirect effects produced by injections with ascitic fluid. Animals consuming this diet exhibited improvements in placental tissue health, leading to increased foetal numbers and enhanced expression of genes involved in placental cell signalling.

Foetal growth is primarily determined by nutrient availability, which is related to the capacity of the placenta to transport nutrients [29, 30]. Foetal macronutrient requirements for oxidative metabolism and growth are met by placental transport, a process that is greatly influenced by the maternal bloodstream and by placental metabolism. The foetal nutrient supply is also considerably affected by the conversion of glucose to lactate (or fructose in some species) in the placenta and by the extensive transamination of amino acids. Placental capacity for nutrient transport increases with foetal demand. As such, maternal-foetal transport kinetics are tightly associated with the expression and distribution of specific transporters among placental cell types and with placental antioxidant response capacity against damage [17] or reduced nutritional supply [29]. Abnormal placental functioning affects foetal growth, especially during late gestation [29–34]. Here, in pregnant, tumour-bearing rats, the consequences of tumour evolution were demonstrated. These included decreased body weight and deleterious effects on foetal growth. These changes suggest that the nutritional supply to the placenta and foetus was diverted to benefit tumour growth. Indirect tumour effects were assessed using an ascitic fluid injection model. This enabled the exclusion of nutritional factors from the observed placental and foetal damage and reinforced that substances produced by tumour and/or host cells can jeopardise the placenta and foetal development.

Several reports have suggested that placental oxidative stress is involved in the etiopathogenesis of pregnancy-related diseases, such as preeclampsia, as well as in foetal growth restriction [17, 29, 35–37]. Reduced perfusion as a result of abnormal placentation leads to ischemic injury and to increased oxidative stress in preeclampsia [35, 36]. The activity of GST, an important detoxification enzyme related to reactive oxygen species processing, can greatly contribute to both foetal and placental antioxidative responses. Consistent with this fact, the activity of this enzyme increased in the groups inoculated with ascites fluid and in the groups supplemented with a leucine-rich diet (L, WL and LA). This result suggests that placental tissue and foetal development were improved. In a previous study, MDA and xanthine oxidase (XO) levels were higher in maternal plasma, umbilical cord plasma, and placental tissues of patients with intrauterine growth restriction (IUGR) than in a control group [17, 35, 36]. Conversely, patients with IUGR exhibited lower antioxidant levels in maternal plasma, umbilical cord plasma, and placental tissues [36]. This reduction suggests that oxidative stress increases in patients with IUGR [35, 38]. As cancer also increases oxidative stress in host tissues [17, 23], in the current study, we inferred that oxidative stress-related damage in placental tissue resulted from tumour growth effects. The observed increases in foetal resorption and reductions in foetal weight likely resulted from decreased placental antioxidative responses. In the W group, increased lipid peroxidation was also observed. Additionally, AP activity, which is generally related to cellular activity and cellular transport, was significantly reduced in the W group. This reduction suggests that tumour growth has deleterious effects on pregnancy. Conversely, placental dysfunction associated with increased lipid peroxidation (MDA) was significantly reduced in the groups fed a leucine-rich diet (L, LW and LA). This reduction was likely associated with the observed positive responses and improvements in foetal and placenta parameters in these groups [17, 20, 28].

Placental function is regulated by numerous factors of foetal, maternal, and placental origin [34, 39–43]. These factors may impact the placenta itself in an autocrine/paracrine manner by integrating numerous and sometimes divergent intracellular regulatory signalling pathways [34, 41–43]. Moreover, changes in placental nutrient transport capacity or supply and/or placental cell activities may not be the primary factors responsible for altered foetal growth, as we verified here in the W group. Other factors could be involved in this harmful process, as evidenced by the similar alterations observed in the A group and the W group. Thus, the most important factor influenced by tumour evolution may be the regulation of placental transport function by maternal and foetal signalling molecules and placental cell signalling. It is important to note that the placenta produces many cytokines and hormones that can act in a paracrine or autocrine manner. These compounds can be affected by humoural factors in ascitic fluid and likely jeopardise placental activity, inducing foetal damage. However, in the A group, several tumour effects were either minimised or abolished, suggesting that placental cell activity can be specifically regulated and that regulatory factors may have opposing effects on different placental process, such as placental transport and nutrient provision to the foetus. Thus, the integration of multiple stimuli is critical for adjusting placental function to accommodate maternal and foetal welfare.

Placental trophoblast cells must integrate numerous and possibly divergent maternal and foetal stimuli and modify cellular functioning according to the host-environment interaction, as we have previously reported [17, 28]. Previous studies have demonstrated that placental signalling requires the integration of multiple pathways, including the regulation of nutrient transport and the regulation of the mTOR signalling pathway, which is the main pathway regulating placental amino acid transport [39–43]. Additionally, alterations in the activity and/or expression of placental mTOR and PPARγ have been observed in human pregnancies complicated by altered foetal growth [31, 42]. It is likely that the reduced foetal growth and increased foetal resorption observed in tumour-bearing rats had multifactorial causes and were potentially associated with decreases in the activities of cell signalling proteins. The above changes are likely affected by placental protein synthesis and degradation, two processes that can be differentially affected by tumour factors (such as proteolysis-inducing factor (PIF) or Walker factor (WF)) to cause foetal impairment in different manners [31, 34]. The mTOR/eIF2 signalling pathway controls protein synthesis in response to nutrient availability. Moreover, mTOR is a positive regulator of placental nutrient transport and is involved in the regulation of foetal growth. These functions are consistent with the results reported here: the levels of mTOR, p70S6K1 and other related proteins decreased in the W group, suggesting failure in placental delivery of nutrition. Conversely, other regulatory processes related may have been responsible for the foetal impairment observed in group A independent of nutrient supply.

The inoculation of ascitic fluid and the effects of humoural factors mimicked the results of PIF and WF in tumour-bearing rats [44]. We previously reported that WF significantly alters protein metabolism and induces foetal damage [2, 11, 44]. It is reasonable to infer that the more severe effects of tumour growth, whether direct or indirect, result from the activation of the proteolytic pathway in placental tissue (increased MuRF-1 and ubiquitin gene and protein expression), especially in the W group and, to a lesser extent, in the A group. This process is responsible for placental failure and consequent foetal damage.

Our study highlights the important role of a leucine-rich diet in modulating placental cell activity and maintaining antioxidative response during tumour growth. As many other studies have proposed, leucine, one of the three branched-chain amino acids, can act as a cellular signal to increase protein synthesis downstream of mTOR, p70S6K1 and eIF4G in skeletal muscle [45–47]. In a previous report, we also demonstrated that consumption of a leucine-rich diet enhances protein synthesis in muscle. Moreover, leucine can improve cellular responses to antioxidative stress and reduce proteolysis by inhibiting the ubiquitin-proteasome pathway [27, 48–51]. Despite foetal weight loss, the tumour-bearing group that received leucine nutritional supplementation exhibited improvements in foetal number and resorption as well as in placental tissue, the most crucial factor in maintaining a normal pregnancy course.

As in previous studies in our laboratory, we found that tumour growth promotes intense mobilisation of muscle proteins associated with diminished protein synthesis [25–27]. Tumour-induced placenta damage was related to changes in key protein-synthesis proteins and increased proteolysis. Similar to other studies, nutritional leucine supplementation did not prevent all harmful effects on foetal development, but it did minimise some of the harmful effects on placental cell activity, enhance protein synthesis and reduce placental proteolysis caused by the direct and indirect effects of tumour growth. However, consistent with increased foetal numbers and reduced foetal absorption rates, alterations in protein synthesis and degradation were observed, as reflected by placental protein balance. This demonstrates that tumour growth (both directly, as in groups W and WL, and indirectly, as in groups A and LA) promotes important changes in the maternal-foetal unit, and these changes can be ameliorated with nutritional leucine supplementation.

## Conclusions

In the current study, we demonstrated that dietary leucine supplementation enhances placental cell signalling, ameliorates deleterious alterations in DNA and protein content, and increases placental cell number. Leucine supplementation also improved the balance between protein synthesis and degradation in the placenta. This was reflected in enhanced cell signalling, including signalling via the mTOR/eIF pathway. Additional experiments are being conducted to better understand the effects produced by ascitic fluid injection and to determine the mechanisms by which leucine improves placental metabolism and counteracts the effects of tumour growth.
